# Agreement between single and multiple ROI strategies for hepatic T1 and ECV mapping in tetralogy of Fallot and Fontan circulation

**DOI:** 10.1007/s00261-026-05441-0

**Published:** 2026-03-09

**Authors:** Adriana Macintyre Innocenzi, Fernanda Padrão Fernandes, José Carlos Pizzolante Secco, Igor Costa Costermani, José Lucas Rebelo, Aline Silva de Medeiros, Daniella Braz Parente, Renata Mello Perez, Ronir Raggio Luiz, Gabriel Cordeiro Camargo, Renata Moll-Bernardes, Mariana Póvoa-Corrêa

**Affiliations:** 1 D’Or Institute for Research and Education, Rio de Janeiro, Brazil; 2 Instituto Nacional de Cardiologia, Rio de Janeiro, Brazil; 3 Federal University of Rio de Janeiro, Rio de Janeiro, Brazil

**Keywords:** Magnetic resonance imaging, Tetralogy of fallot, Fontan procedure, Fibrosis, Liver diseases

## Abstract

**Purpose:**

Native T1 and extracellular volume (ECV) mapping have emerged as promising tools for noninvasive assessment of hepatic congestion and fibrosis; however, optimal strategies for region-of-interest (ROI) selection remain uncertain. The aim was to evaluate the level of agreement between two methods for quantifying hepatic native T1 and ECV: single wide ROI versus multiple small ROIs, in patients with repaired tetralogy of Fallot (TOF) and Fontan circulation.

**Methods:**

This agreement study prospectively enrolled outpatients with repaired TOF or Fontan circulation between 2022 and 2025. All participants underwent hepatic evaluation using a cardiac Magnetic Resonance Imaging (MRI) protocol on a 3.0-T scanner. Native and post-contrast T1 mapping were acquired using Modified Look-Locker Inversion Recovery (MOLLI) sequences. Hepatic T1 and ECV were measured using (1) one wide ROI avoiding vascular structures and (2) three small circular ROIs placed in distinct parenchymal regions. Agreement was assessed using intraclass correlation coefficients (ICC) and survival-agreement plots.

**Results:**

A total of 61 patients was included (31 with TOF; 30 with Fontan circulation). Agreement between single-ROI and multiple-ROI strategies was excellent in TOF patients for both native T1 and ECV (ICC = 0.98), and good in patients with Fontan circulation for both parameters (ICC = 0.79). Survival-agreement analysis demonstrated that in TOF patients, all measurements differed by less than 80 ms for native T1 and less than 5% for ECV. In Fontan patients, 86% of measurements showed differences < 80 ms for native T1 and < 5% for ECV, reflecting greater hepatic heterogeneity while preserving overall concordance between ROI strategies.

**Conclusions:**

Hepatic native T1 and ECV measurements showed excellent agreement between single-wide and multiple-small ROI strategies in repaired TOF, supporting interchangeable use for longitudinal follow-up. In Fontan patients, concordance between methods was also good, as demonstrated by both ICC and survival-agreement analyses, indicating that either strategy remains feasible; nonetheless, the greater hepatic heterogeneity in this population warrants some caution when comparing measurements obtained with different ROI approaches.

**Graphical abstract:**

**Figure:**
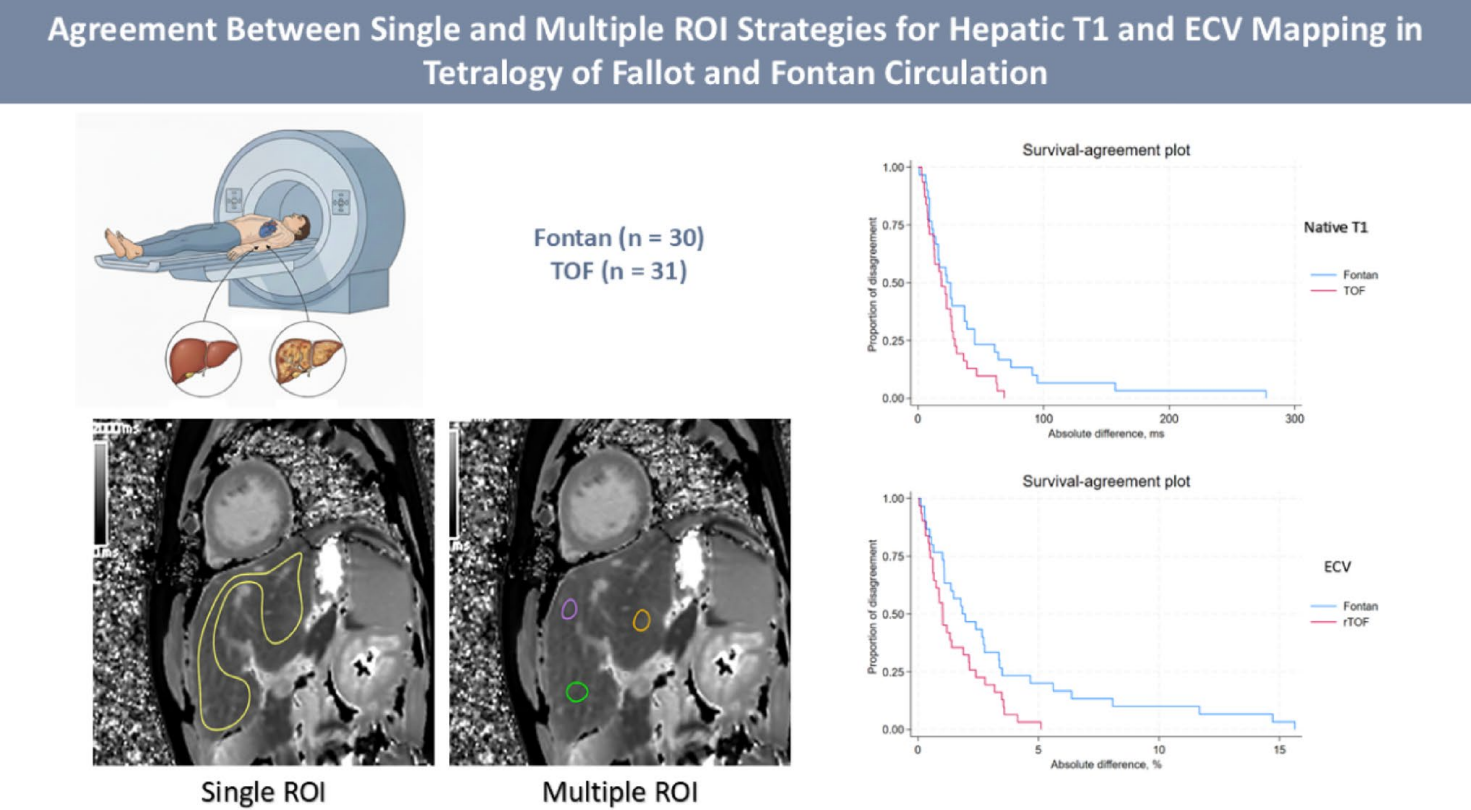

## Introduction

As survival improves in congenital heart disease (CHD), growing evidence shows that chronically elevated venous pressures can lead not only to cardiac sequelae but also to clinically relevant hepatic involvement, which is increasingly recognized as a major end-organ complication [1]. Among CHD physiologies, the Fontan circulation and repaired tetralogy of Fallot (TOF) represent two distinct conditions that may impose chronic hemodynamic stress on the liver [2, 3].

The Fontan procedure, used in single-ventricle physiology, characterized by passive systemic venous return to the pulmonary arteries in the absence of a subpulmonary pump, inherently predisposes patients to chronic hepatic venous congestion [2]. In contrast, individuals with repaired TOF, despite biventricular physiology, may develop persistent right ventricular volume or pressure overload due to residual pulmonary regurgitation or stenosis [4]. In both physiologic settings, prolonged venous congestion may lead to congestive hepatopathy, progressive parenchymal remodeling, and ultimately liver fibrosis [5–8].

Assessment of hepatic fibrosis is clinically relevant as liver disease directly affects clinical management, prognosis, and eligibility for heart transplantation [3, 9–11]. Multiparametric hepatic magnetic resonance imaging, particularly native T1 and extracellular volume (ECV) mapping, has emerged as a promising noninvasive technique for estimating the degree of fibrosis [12, 13]. These parameters reflect tissue alterations associated with chronic injury, persistent venous congestion, and interstitial fibrotic remodeling [14, 15].

Several methodologies for hepatic native T1 quantification have been described, ranging from a single large region of interest (ROI) encompassing a broad parenchymal area to smaller ROIs positioned in distinct regions [14, 16, 17]; yet, no consensus exists regarding the most accurate or reproducible technique. Due to the spatial heterogeneity of hepatic involvement, especially in Fontan patients, differences in ROI size and distribution may affect hepatic T1 and ECV measurements, underscoring the importance of validating agreement between ROI strategies to ensure reproducibility and clinical applicability.

The aim of this study was to evaluate the agreement between two ROI strategies for quantifying hepatic native T1 and ECV, using the same MRI acquisition protocol applied for cardiac assessment, in patients with repaired tetralogy of Fallot and Fontan circulation.

## Materials and methods

### Study design

This agreement study included the prospective and consecutive recruitment of outpatients with a diagnosis of repaired tetralogy of Fallot (TOF) or Fontan circulation who were followed at specialized cardiology clinics between 2022 and 2025. Patients presenting standard contraindications to magnetic resonance imaging were excluded.

### MRI protocol

All participants underwent hepatic evaluation using a cardiac magnetic resonance protocol on a 3.0-T scanner (Magnetom Prisma; Siemens Healthcare, Erlangen, Germany). Examinations were performed after a 2 to 4-hour fasting period and had an approximate duration of 50 min. Peripheral venous blood sampling for hematocrit determination was performed on the same day as the MRI.

Native and post-contrast T1 mapping were performed using modified Look-Locker inversion recovery (MOLLI) sequences with single-shot SSFP readouts, as originally described by Messroghli et al. [18]. A 5(3)3 MOLLI scheme was used for both native (pre-contrast) and post-contrast acquisitions. ECG-gated T1-mapping was performed using a sagittal slice positioned at the anatomical level of the mid-ventricular short-axis plane, in which both the heart and the adjacent hepatic parenchyma are simultaneously visualized, ensuring consistent liver sampling, with the following parameters: TR, 280.6 ms (long); TR, 360.6 ms (short); TE 1.12 ms; flip angle, 35°; voxel size, 1.4 mm × 1.4 mm × 8.0 mm; acceleration factor, 2. Standard motion correction was applied. Native hepatic T1 values were measured before contrast administration, and post-contrast T1 values were acquired ≥ 15 min after intravenous gadolinium, allowing adequate contrast equilibrium within the extracellular space [18].

A gadolinium-based contrast agent (Gd-DOTA, Dotarem; Guerbet) was administered intravenously at 0.2–0.3 mmol/kg, and post-contrast T1-weighted maps were acquired approximately 15 min after injection, according to standard extracellular volume fraction (ECV) assessment recommendations [13].

T1 maps were generated inline by the MRI scanner using the vendor-provided reconstruction pipeline; therefore, curve fitting and bias correction were inherent to the scanner implementation.

### ROI definition and image analysis

Hepatic and intracardiac blood-pool ROIs were manually delineated for quantitative T1 analysis by a single experienced radiologist for the full study cohort. Interobserver agreement was subsequently assessed in a representative subset, as described below. The use of one large and three small ROIs was based on prior hepatic T1 mapping studies [17] which adopted distributed sampling to improve spatial representation and reduce the impact of focal heterogeneity and vascular contamination. We used two contrasting ROI strategies:

1. Single large ROI, encompassing the largest homogeneous parenchymal area while excluding major vessels.
2. Multiple small ROIs: three small circular ROIs (each approximately 0.8–1.0 cm²) were manually placed in spatially separated visually homogeneous regions of the hepatic parenchyma within the same sagittal slice used for cardiac short-axis imaging. Minor variation in ROI size was permitted to accommodate local anatomical constraints while avoiding vessels, biliary structures, and areas of signal heterogeneity, and maintaining a minimum distance of approximately 2 mm from anatomical boundaries. This approach aimed to provide spatially representative sampling of the liver parenchyma.

ROIs were consistently drawn on the same T1-mapping slice acquired at the level of the mid-ventricular short-axis, which corresponds to a sagittal imaging plane displaying both the heart and the left hepatic lobe, in both native and post-contrast acquisitions, ensuring standardized anatomical positioning across participants. The intracardiac blood-pool ROI was drawn in the center of the left ventricle, completely excluding myocardial tissue.

Pixel intensity values and T1 relaxation times were extracted to quantify native and post-contrast hepatic T1 and to compute hepatic ECV. Image review and general processing were performed using OsiriX MD software (version 12.0.3; Pixmeo SARL, Bernex, Switzerland), whereas quantitative hepatic analyses, including T1 and ECV measurements, were carried out using Circle CVI42 software (version 5.14.2; Circle Cardiovascular Imaging Inc., Calgary, AB, Canada).

### Statistical analysis

Continuous variables are presented as means ± standard deviations, and categorical variables as absolute and relative frequencies.

Interobserver agreement was evaluated in a representative subset of 35 examinations, selected to include both diagnostic groups (repaired TOF and Fontan circulation) and to span the full range of hepatic native T1 and ECV values observed in the overall cohort, including cases with greater parenchymal heterogeneity. ROI placement and measurements were independently performed by two experienced radiologists (each with more than 10 years of experience in MRI). Interobserver reliability was assessed using a one-way random-effects model for absolute agreement and quantified by the single-measure intraclass correlation coefficient (ICC). In addition, Bland–Altman analysis was performed to estimate interobserver mean bias and 95% limits of agreement. The interobserver analysis was conducted solely to assess reader-related variability; therefore, the mean of the two readers was not used for subsequent analyses.

Agreement between ROI strategies was assessed using the ICC, estimated from a two-way mixed-effects model assuming absolute agreement. ICC values > 0.90 were considered excellent, and 0.75–0.90 good, according to Koo and Li [19]. Agreement was further evaluated using Bland–Altman analysis, estimating mean bias and 95% limits of agreement, with visual assessment for proportional bias and heteroscedasticity, performed separately for TOF and Fontan cohorts. In addition, agreement across increasing tolerance thresholds was explored using the survival-agreement plots as described by Luiz et al. [20] which display the proportion of subjects whose measurement differences exceed progressively larger tolerance thresholds, allowing visualization of how robust the agreement between two methods remains across clinically meaningful ranges. Post-contrast T1 was measured solely for ECV computation and is therefore not reported as an independent outcome.

A two-sided p-value < 0.05 was considered statistically significant. All statistical analyses were performed using Stata software, version 19 (StataCorp LLC, College Station, TX).

## Results

In the TOF group (*n* = 31), the mean age was 12.8 ± 3.3 years, and 10 (32.3%) were female. Participants had a mean weight of 50.6 ± 16.7 kg and a BMI of 20.5 ± 3.5 kg/m². The right ventricular indexed end-diastolic volume (EDVI) averaged 113.0 ± 26.1 mL/m², and the right ventricular ejection fraction was 51.7 ± 4.8% (Table 1).

In the Fontan group (*n* = 30), the mean age was 17.3 ± 5.0 years, with 19 (63.3%) female participants. Mean weight and BMI were 51.2 ± 14.7 kg and 20.6 ± 3.8 kg/m², respectively. The dominant-ventricle EDVI was 87.7 ± 19.3 mL/m², and the ejection fraction averaged 57.0 ± 8.6% (Table 1).

**Fig. 1:**
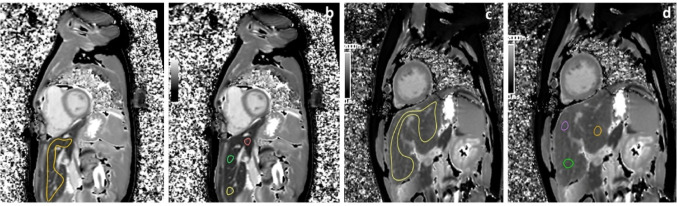
T1 maps of patients with repaired TOF (**a**,** b**) and with Fontan circulation (**c**,** d**). Sagittal MRI slices demonstrating two measurement approaches: a single wide ROI positioned over the largest homogeneous region of the hepatic parenchyma (**a**,** c**); and multiple small circular ROIs distributed across different homogeneous areas of the hepatic parenchyma (**b**,** d**). *TOF* tetralogy of Fallot, *MRI* magnetic resonance imaging, *ROI* region of interest

In the TOF group, mean native T1 was 884 ± 86.3 ms using the multiple-ROI method and 898.7 ± 88.0 ms using the single-ROI method. In the Fontan group, native T1 values were 966.6 ± 84.7 ms and 989.4 ± 75.6 ms using multiple-ROI and single-ROI methods, respectively (Fig. 1).

Hepatic ECV in the TOF group was 37.2 ± 6.2% using the multiple-ROIs method and 38.2 ± 6.4% using the single-ROI method. In the Fontan group, hepatic ECV measured 41.2 ± 5.6% with the multiple-ROIs approach and 42.1 ± 7.0% with the single-ROI method.

### Interobserver agreement

To establish baseline reader-to-reader variability, interobserver reliability was evaluated in a representative subset of 35 examinations. Excellent agreement was observed for both ROI strategies and for both native and post-contrast T1 measurements. For the single large ROI approach, interobserver agreement was excellent for native T1 (ICC = 0.96; 95% CI: 0.93–0.98; *p* < 0.001) and for post-contrast T1 (ICC = 0.98; 95% CI: 0.96–0.99; *p* < 0.001). For the multiple small ROI strategy, agreement was similarly excellent for native T1 (ICC = 0.94; 95% CI: 0.88–0.97; *p* < 0.001) and post-contrast T1 (ICC = 0.99; 95% CI: 0.97–0.99; *p* < 0.001).

**Fig. 2:**
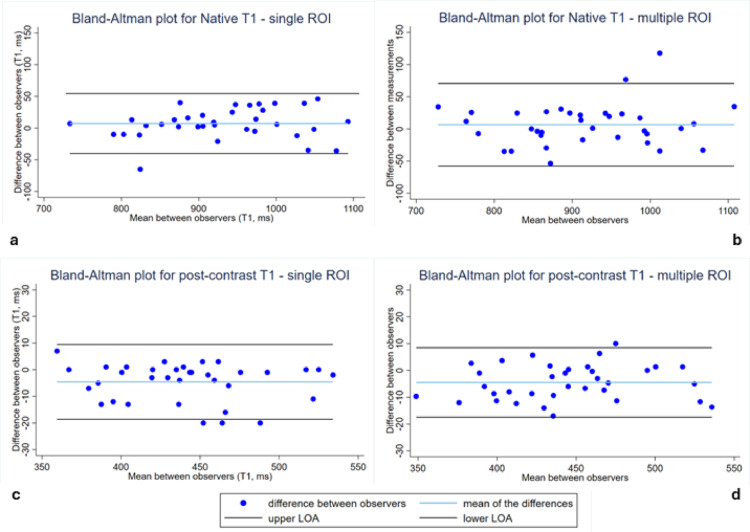
Bland–Altman plots illustrating interobserver agreement between the single-ROI and multiple-ROI strategies. Panels show **a** native T1 using single-ROI strategy, **b** native T1 using multiple-ROI strategy, **c** post-contrast T1 using single-ROI strategy, and **d** post-contrast T1 using multiple-ROI strategy. Solid horizontal blue line represents mean bias, and horizontal black lines indicate the 95% limits of agreement. Both ROI strategies demonstrate small mean bias and comparable dispersion, with no visual evidence of proportional bias. *LOA* limit of agreement, *ROI* region of interest

Bland–Altman analysis demonstrated small interobserver mean bias with comparable dispersion between strategies. For native T1, mean bias was 7.11 ms for the single ROI strategy (95% limits of agreement: −40.13 to + 54.35 ms) and 6.49 ms for the multiple ROI strategy (95% limits of agreement: −57.68 to + 70.66 ms). For post-contrast T1, mean bias was − 4.57 ms (95% limits of agreement: −18.62 to + 9.48 ms) for the single ROI approach and − 4.48 ms (95% limits of agreement: −17.46 to + 8.50 ms) for the multiple ROI approach. No systematic proportional bias was visually detected. These findings provide a quantitative reference for expected reader-related variability and confirm robust reproducibility for both ROI strategies (Fig. 2).

### Agreement analysis

Agreement between single-ROI and multiple-ROI strategies was first assessed using ICC. In patients with repaired TOF, agreement was excellent for both native T1 (ICC = 0.978; 95% CI: 0.955–0.990; *p* < 0.001) and ECV (ICC = 0.978; 95% CI: 0.955–0.990; *p* < 0.001). In Fontan patients, agreement was good for both native T1 (ICC = 0.791 (95% CI: 0.560–0.900; *p* < 0.001) and ECV (ICC = 0.790 (95% CI: 0.562–0.900; *p* < 0.001).

**Table 1:** Clinical and imaging characteristics of the TOF and Fontan groups

Parameter	TOF (*n* = 31)	Fontan (*n* = 30)
Age (years)	12.8 ± 3.3	17.3 ± 5.0
Female sex, n (%)	10 (32.3%)	19 (63.3%)
Weight (Kg)	50.6 ± 16.7	51.2 ± 14.7
BMI (kg/m²)	20.5 ± 3.5	20.6 ± 3.8
EDVI (mL/m²)	113.0 ± 26.1*	87.7 ± 19.3†
Ejection fraction (%)	51.7 ± 4.8	57.0 ± 8.6

Values are presented as mean ± standard deviation or as absolute counts (percentages).

*BMI* body mass index, *EDVI* indexed end-diastolic volume, *TOF* tetralogy of Fallot

*Right ventricle; †Dominant ventricle

**Table 2:** Hepatic native T1 and extracellular volume values stratified by measurement technique

Parameter	Method	TOF (*n* = 31)	Fontan (*n* = 30)
Native T1 (ms)	Multiple-ROIs	884.2 ± 86.3	966.6 ± 84.7
	Single-ROI	898.7 ± 88.0	989.4 ± 75.6
ECV (%)	Multiple-ROIs	37.2 ± 6.2	41.2 ± 5.6
	Single-ROI	38.2 ± 6.4	42.1 ± 7.0

Values are presented as mean ± standard deviation

*ECV* extracellular volume, *ROI* region of interest, *TOF* tetralogy of Fallot

**Table 3:** Intraclass correlation coefficients assessing agreement between the measurement techniques

Group	Parameter	ICC	95% CI	*p*	Agreement
TOF	Native T1	0.978	0.955–0.990	< 0.001	Excellent
Fontan	Native T1	0.791	0.560–0.900	< 0.001	Good
TOF	ECV	0.978	0.955–0.990	< 0.001	Excellent
Fontan	ECV	0.790	0.562–0.900	< 0.001	Good

*CI* confidence interval, *ECV* extracellular volume, *ICC* intraclass correlation coefficients, *TOF* tetralogy of Fallot

**Fig. 3:**
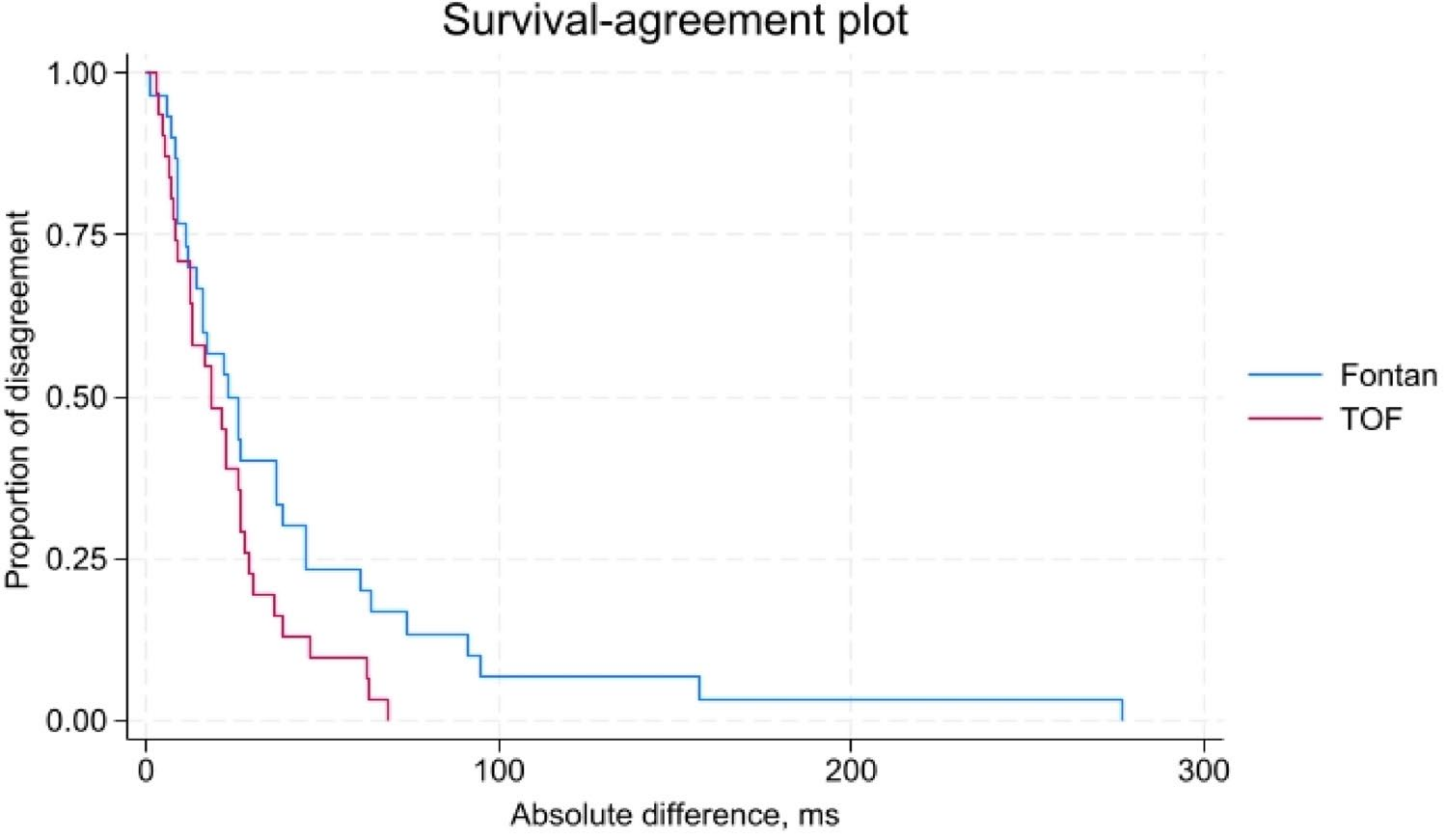
Survival-agreement plot illustrates the cumulative proportion of discordant measurements between single and multiple ROI strategies for hepatic native T1 across increasing absolute differences in TOF and Fontan patients, depicting the tolerance thresholds at which complete agreement between methods is observed for each group. In the TOF cohort, all patients showed absolute differences < 80 ms, whereas in Fontan group, 86% of patients showed differences < 80 ms. *ROI* region of interest, *TOF* tetralogy of Fallot

A survival-agreement plot was generated to present the proportion of discordant observations between the two hepatic native T1 measurement techniques across increasing absolute differences. In the TOF group, 50% discordance was reached at an absolute difference of approximately 20 ms, and discordance declined to zero at a tolerance limit of about 80 ms. In the Fontan group, 50% discordance occurred at roughly 40 ms, 86% of patients showed differences < 80 ms, and the curve reached zero discordance only at differences close to 250–300 ms. The complete agreement curves for both groups are shown in Fig. 3.

**Fig. 4:**
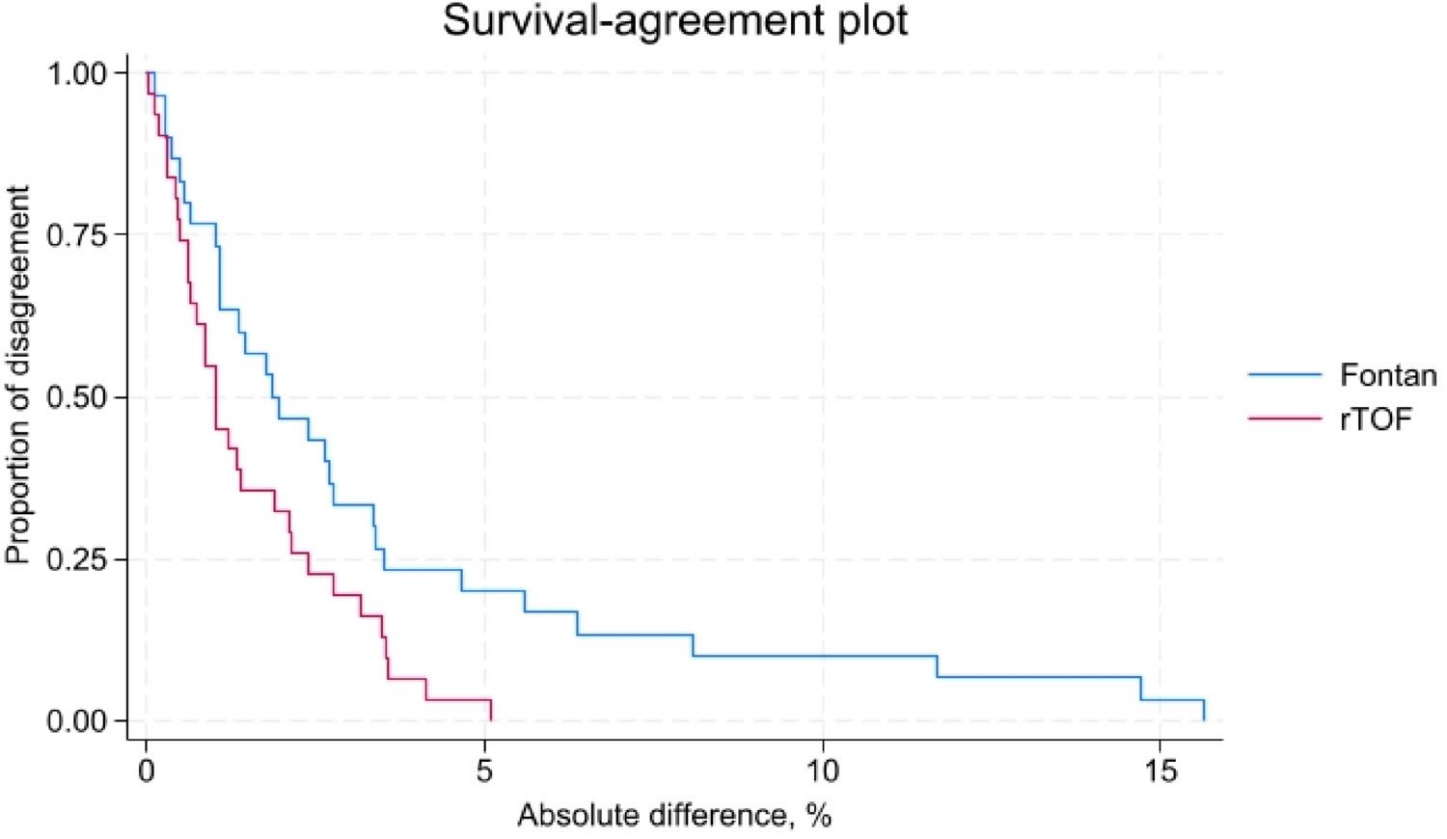
Survival-agreement plot illustrates the cumulative proportion of discordant measurements between single and multiple ROI strategies for hepatic ECV across increasing absolute differences in TOF and Fontan patients, depicting the tolerance thresholds at which complete agreement between methods is observed for each group. In the TOF cohort, approximately almost all patients (97%) demonstrated absolute differences < 5%, whereas in Fontan patients, 86% of patients fell within this range. *ROI* region of interest, *ECV* extracellular volume, *TOF* tetralogy of Fallot

Similarly, a survival-agreement plot was constructed to display the proportion of discordant observations between the two ECV measurement techniques across increasing absolute percentage differences. In the TOF group, 50% discordance occurred at an absolute difference of approximately 2%, and the curve reached zero discordance at about 5%. In the Fontan group, 50% discordance was observed at a difference of roughly 3–4%, 86% of patients showed a discordance < 5%, with zero discordance occurring only at tolerance limits close to 15%. The complete agreement curves for both groups are presented in Fig. 4.

### Bland–Altman analysis

**Fig. 5:**
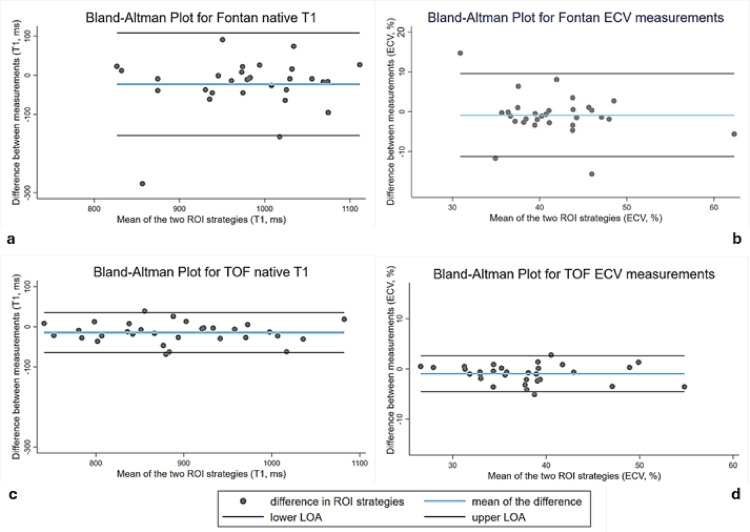
Bland–Altman plots illustrating agreement between the single-ROI and multiple-ROI strategies. Panels show **a** native T1 in patients with repaired TOF, **b** ECV in TOF, **c** native T1 in Fontan patients, and **d** ECV in Fontan patients. Solid horizontal blue line represents mean bias, and horizontal black lines indicate the 95% limits of agreement. In TOF, both native T1 and ECV demonstrate small mean bias and narrow limits of agreement, indicating good agreement. In Fontan patients, dispersion is greater, particularly for native T1, reflecting increased hepatic heterogeneity, although no proportional bias or heteroscedasticity is observed. *ECV* extracellular volume, *LOA* limit of agreement, *ROI* region of interest, *TOF* tetralogy of Fallot

In patients with repaired TOF, Bland–Altman analysis demonstrated good agreement between the single-ROI and multiple-ROI strategies for both native T1 and ECV measurements. For native T1, a small systematic bias was observed, with a mean difference of 14.5 ms and 95% limits of agreement ranging from approximately − 65 to + 40 ms, indicating moderate to good agreement despite a few individual discrepancies. For ECV, mean bias was low at 0.96%, with 95% limits of agreement between approximately − 4.5% and + 2.5%, reflecting good agreement between sampling strategies. No proportional bias or heteroscedasticity was visually detected for either parameter, supporting the absence of clinically relevant systematic measurement error across the range of values (Fig. 5a and b).

In Fontan patients, Bland–Altman analysis demonstrated overall moderate agreement between ROI strategies, with greater dispersion compared with the TOF cohort. For native T1, mean bias was small (− 22.77 ms), with relatively wide 95% limits of agreement (− 153.73 to + 108.20 ms) and a few outliers, consistent with the pronounced hepatic heterogeneity inherent to univentricular physiology. Similarly, for ECV, mean bias was small (− 0.85%), but limits of agreement remained moderately wide (− 11.26% to + 9.56%), with most observations lying within these bounds. No proportional bias or heteroscedasticity was visually detected for either parameter, although occasional individual discrepancies were observed (Fig. 5c and d).

## Discussion

In this study, hepatic native T1 and ECV measurements obtained using a single wide ROI and multiple small ROIs demonstrated strong overall agreement, although distinct patterns were observed between the TOF and Fontan populations. These different patterns are easily observed in the survival-agreement analysis. In the TOF cohort, ICC values were excellent and accompanied by narrow confidence intervals, indicating very high concordance and supporting the practical interchangeability of single-ROI and multi-ROI strategies in this group. Nevertheless, while agreement in the Fontan cohort remained statistically good, the wider confidence intervals suggest greater variability and reduced certainty regarding the equivalence of the two ROI approaches. Similar findings were observed with Bland-Altman analysis. This likely reflects the increased spatial heterogeneity of hepatic parenchyma in patients with univentricular physiology, where chronic venous congestion, sinusoidal dilatation, and regionally variable fibrotic remodeling contribute to less uniform tissue characteristics and greater sensitivity to ROI positioning [13, 21].

Interobserver analysis demonstrated excellent reproducibility for both ROI strategies, with high ICC values and small mean bias on Bland–Altman analysis, indicating low reader-related variability. Importantly, the magnitude of variability observed between single-ROI and multiple-ROI strategies was small and generally comparable to, or only slightly greater than, expected interobserver variability, suggesting that differences between sampling approaches are limited in magnitude and do not substantially exceed normal reader-related dispersion. Notably, interobserver Bland-Altman analyses did not demonstrate a consistent advantage of the multiple-ROI strategy over the single-ROI approach; visual inspection of the plots showed comparable, and in some instances slightly narrower, limits of agreement with the single-ROI strategy for both native and post-contrast T1 measurements, with no systematic reduction in interobserver variability observed when multiple ROIs were used.

The incorporation of the survival-agreement analysis, a graphical method originally proposed by Luiz et al. [20] to evaluate agreement based on the proportion of discordant observations across increasing tolerance limit, provided an important added dimension to our assessment of concordance between ROI strategies. Unlike the ICC, which offers a single global estimate of agreement influenced by overall sample variability, the survival-agreement approach allowed us to visualize the entire distribution of differences between methods across progressively stricter tolerance limits. This graphical representation was particularly informative in the Fontan group, where hepatic parenchyma is more heterogeneous. In this cohort, the survival-agreement curves declined more gradually, clearly illustrating that discordance becomes more prominent as tolerance thresholds narrow, an insight not fully captured by the ICC alone. Even at the most restrictive tolerance thresholds, the majority of Fontan cases (86%) continued to exhibit agreement between methods, highlighting that, despite greater parenchymal heterogeneity, the two ROI strategies remain largely concordant.

Our results align with an increasing body of evidence supporting the technical reliability and clinical relevance of hepatic parametric mapping in congenital heart disease. De Lange et al. [16] demonstrated excellent intra- and interobserver reproducibility of hepatic T1 and ECV in pediatric patients with single-ventricle physiology and emphasized that averaging values from multiple ROIs distributed across the liver provides the most representative assessment, particularly in the presence of spatial heterogeneity. Similarly, Kazour et al. [14], studying cohorts including repaired TOF, Fontan patients, and healthy controls, adopted a multi-ROI approach as standard methodology, highlighting the importance of distributed sampling to minimize focal heterogeneity and avoid inadvertent inclusion of vascular structures. Nevertheless, our study showed strong concordance, thereby supporting the practical equivalence of single-ROI and multi-ROI methods for patients with TOF. ROI placement time was not formally recorded in this study; however, in routine practice, the time required for drawing a single large ROI and three spatially distributed small ROIs appears comparable. Although a single large ROI may seem simpler, careful exclusion of vascular structures and heterogeneous areas can require similar attention.

Ide et al. [8] demonstrated significant spatial variability in hepatic T1 values across Couinaud-defined segments, particularly with higher values near the liver margins and adjacent to the inferior vena cava, reinforcing that regional heterogeneity is an intrinsic feature of congestive hepatopathy rather than a measurement artifact, corroborating our findings of wider confidence interval for native T1 and ECV in patients with univentricular physiology. Yanovskiy et al. [22] further corroborated this by demonstrating marked differences between central and peripheral hepatic zones in univentricular patients, supporting the concept that spatial variability reflects heterogeneous fibrotic remodeling and congestion patterns in advanced disease.

Importantly, the high concordance between ROI strategies allows reliable comparison of examinations performed at different time points or across centers, even when different measurement approaches have been applied, thereby supporting longitudinal and multicenter consistency in liver T1 mapping studies. In addition, the interobserver agreement was high, supporting the robustness of the measurements. These data strengthen the evidence supporting hepatic T1 and ECV as reliable noninvasive biomarkers of liver involvement in congenital heart disease, while highlighting that disease severity and parenchymal heterogeneity should inform methodological choices.

Our findings align with growing evidence that hepatic parametric mapping is both technically feasible and methodologically robust across congenital heart disease populations [5]. Despite these differences in sampling strategies, a consistent theme across studies is that hepatic T1 mapping behaves as a stable biomarker when ROIs are systematically positioned in morphologically homogeneous parenchyma and vascular structures are carefully avoided [14, 16].

### Clinical and methodological implications

The potential synergistic burden of congenital heart disease and liver disorders, including metabolic dysfunction–associated steatotic liver disease [23], underscores the importance of early identification, longitudinal surveillance, and integrated preventive strategies to mitigate progressive hepatic injury in this growing and vulnerable population. Within this clinical context, the high agreement between single-ROI and multi-ROI approaches offers important practical advantages, enabling reliable comparison of measurements across serial examinations and multicenter studies. This methodological robustness facilitates data harmonization and pooling of results, while supporting consistent clinical interpretation and decision-making, provided that acquisition parameters remain standardized, particularly in the absence of marked parenchymal heterogeneity.

The findings suggest that increased parenchymal heterogeneity may be associated with greater dispersion between sampling strategies. Although this study was not designed to establish decision thresholds, future work may explore whether intra-ROI variability may help identify cases in which more spatially distributed sampling is advantageous.

### Strengths and limitations

Limitations include the single-center nature of the study, and lack of histological correlation, which precluded our ability to determine the optimal strategy, nevertheless, defining the best sampling approach was not our primary objective. Hepatic T1 measurements were obtained using a fixed 5(3)3 MOLLI scheme, which is known to be influenced by heart rate; although this may affect absolute T1 values, identical acquisition parameters were applied across all examinations, mitigating its impact on the comparative agreement between ROI strategies. Post-contrast T1 mapping was not optimized for short T1 values, which may affect absolute accuracy but is unlikely to impact the comparative agreement analyses. Additionally, although both ROI strategies performed well within our controlled conditions, variation in scanner platform and sequence implementation may influence reproducibility in broader clinical practice. As all examinations were performed at 3.0 T, the applicability of our findings to 1.5 T systems could not be assessed, and further studies are warranted to validate agreement between ROI strategies across different field strengths. Strengths of this study include the combined use of ICC and survival-agreement analyses, which provided complementary perspectives on method reproducibility and helped to mitigate the analytical variability introduced by the greater hepatic heterogeneity characteristic of Fontan patients.

## Conclusions

Hepatic native T1 and ECV measurements obtained using single wide ROI and multiple small ROIs demonstrated very high concordance in patients with repaired TOF within the context of our standardized cardiac MRI acquisition protocol supporting the reliable use of either strategy under these conditions. This agreement enables consistent comparison across serial examinations even when different ROI approaches have been used, enhancing the feasibility of longitudinal follow-up of liver involvement. In the Fontan cohort, agreement between ROI strategies was also good, and although the survival-agreement analysis corroborated this concordance, the greater hepatic heterogeneity characteristic of univentricular physiology warrants a more cautious interpretation when different sampling methods are applied.

## Data Availability

The data underlying this study will be made available by the authors upon reasonable request and with appropriate justification.
